# Antioxidant Carbon Nanoparticles Inhibit Fibroblast-Like Synoviocyte Invasiveness and Reduce Disease Severity in a Rat Model of Rheumatoid Arthritis

**DOI:** 10.3390/antiox9101005

**Published:** 2020-10-16

**Authors:** Mark R. Tanner, Redwan Huq, William K. A. Sikkema, Lizanne G. Nilewski, Nejla Yosef, Cody Schmitt, Carlos P. Flores-Suarez, Arielle Raugh, Teresina Laragione, Pércio S. Gulko, James M. Tour, Christine Beeton

**Affiliations:** 1 Department of Molecular Physiology and Biophysics, Baylor College of Medicine, Houston, TX 77030, USA; mrtanner@bcm.edu (M.R.T.); redwanhuq@gmail.com (R.H.); nejlaphd@gmail.com (N.Y.); sa201348@atsu.edu (C.S.); carlos.floressuarez@bcm.edu (C.P.F.-S.); raugh@bcm.edu (A.R.); 2 Interdepartmental Graduate Program in Translational Biology and Molecular Medicine, Baylor College of Medicine, Houston, TX 77030, USA; 3 Graduate Program in Molecular Physiology and Biophysics, Baylor College of Medicine, Houston, TX 77030, USA; 4 Department of Chemistry, Rice University, Houston, TX 77005, USA; william.sikkema@gmail.com (W.K.A.S.); lizanne.nilewski@gmail.com (L.G.N.); 5 Department of Medicine, Division of Rheumatology, Icahn School of Medicine at Mount Sinai, New York, NY 11030, USA; Teresina.laragione@mssm.edu (T.L.); Percio.gulko@mssm.edu (P.S.G.); 6 The NanoCarbon Center, Rice University, Houston, TX 77005, USA; 7 Center for Drug Discovery and Biology of Inflammation Center, Baylor College of Medicine, Houston, TX 77030, USA

**Keywords:** synovial fibroblast, oxidative stress, nanomaterials

## Abstract

Reactive oxygen species have been involved in the pathogenesis of rheumatoid arthritis (RA). Our goal was to determine the effects of selectively scavenging superoxide (O_2_^•−^) and hydroxyl radicals with antioxidant nanoparticles, called poly(ethylene glycol)-functionalized hydrophilic carbon clusters (PEG-HCCs), on the pathogenic functions of fibroblast-like synoviocytes (FLS) from patients with rheumatoid arthritis (RA) and on the progression of an animal model of RA. We used human FLS from patients with RA to determine PEG-HCC internalization and effects on FLS cytotoxicity, invasiveness, proliferation, and production of proteases. We used the pristane-induced arthritis (PIA) rat model of RA to assess the benefits of PEG-HCCs on reducing disease severity. PEG-HCCs were internalized by RA-FLS, reduced their intracellular O_2_^•−^, and reduced multiple measures of their pathogenicity in vitro, including proliferation and invasion. In PIA, PEG-HCCs caused a 65% reduction in disease severity, as measured by a standardized scoring system of paw inflammation and caused a significant reduction in bone and tissue damage, and circulating rheumatoid factor. PEG-HCCs did not induce lymphopenia during PIA. Our study demonstrated a role for O_2_^•−^ and hydroxyl radicals in the pathogenesis of a rat model of RA and showed efficacy of PEG-HCCs in treating a rat model of RA.

## 1. Introduction

Rheumatoid arthritis (RA) is the most common form of inflammatory arthritis and both immune cells and resident joint cells, such as fibroblast-like synoviocytes (FLS) participate in pathogenesis [1,2,3,4,5].

Reactive oxygen species (ROS) such as superoxide (O_2_^•−^) are implicated in the progression of RA [6,7,8,9]. In RA, O_2_^•−^ and hydroxyl radicals are produced in the joints and contribute to their damage. Furthermore, mitochondrial dysfunction in FLS during RA leads to elevated levels of intracellular O_2_^•−^, enhancing the aggressive phenotype of these cells during RA [10,11]. Antioxidants have been proposed as therapies to treat RA though broad antioxidants that target many ROS and nitrogen reactive species are toxic at high doses for prolonged periods of time [12]. There is therefore an unmet need for the development of potent and O_2_^•−^ selective antioxidants as potential therapeutics for RA. 

Poly(ethylene glycol)-functionalized hydrophilic carbon clusters (PEG-HCCs) are nontoxic graphene ribbon nanomaterials and are highly potent and selective scavengers of O_2_^•−^ to the exclusion of any other reactive oxygen or nitrogen species [13,14,15]. PEG-HCCs do not pass radicals onto other molecules, rapidly inactivate, or require assistance from auxiliary molecules. They are hydrophilic, contain no significant trace metals by inductively coupled plasma mass cytometry, and cannot form fibrous aggregates that perturb phagocytes. In studies of O_2_^•−^ quenching, 70 μg of PEG-HCCs had a quenching effect similar to that of 10 U/mg O_2_^•−^ dismutase, making them even more catalytically effective than the overexpression of this enzyme for reducing levels of O_2_^•−^ [14].

We have previously demonstrated that PEG-HCCs are preferentially endocytosed by highly metabolic cells, such as T lymphocytes, and inhibit their function via scavenging intracellular O_2_^•−^ [16,17]. As FLS are highly metabolic and have elevated levels of O_2_^•−^ during RA [10,18], we sought to define their sensitivity to PEG-HCCs and the ability of these nanoparticles to treat a rat model of RA. 

Our results show that PEG-HCCs are internalized by human FLS, scavenge intracellular O_2_^•−^, and inhibit FLS proliferation and invasiveness. In rats with pristane-induced arthritis (PIA), PEG-HCCs are found in the inflamed joints after systemic injection and they reduce arthritis severity and joint damage without inducing lymphopenia. Overall, our study suggests PEG-HCCs as a novel therapeutic option for the treatment of RA by scavenging O_2_^•−^ and inhibiting RA-FLS proliferation and invasiveness.

## 2. Materials and Methods 

### 2.1. PEG-HCCs

The preparation and characterization of PEG-HCCs were completed as earlier described [19,20]. PEG-HCCs were sterile filtered in PBS to a dilution of 1.0 mg/mL and tested for endotoxins prior to use [16].

### 2.2. Animals and Induction and Monitoring of Pristane-Induced Arthritis

Experiments involving rats were conducted under a protocol approved by the Institutional Animal Care and Use Committee at Baylor College of Medicine (AN-4351). Female dark agouti (DA) rats, 8–11 weeks old, were purchased from Envigo (Indianapolis, IN, USA) and provided food and water *ad libitum* in a facility approved by the Association for Assessment and Accreditation of Laboratory Animal Care.

PIA was chosen as a model for this study because it mimics RA in humans in terms of involvement of FLS and immune cells, and production of rheumatoid factor [21]. PIA was induced by the subcutaneous injection of 150 µL pristane (2,6,10,14-tetramethylpentadecane; MP Biomedicals, Irvine, CA, USA) at the base of the tail [22,23,24]. Rats were monitored daily, and clinical scores recorded as follows: 1 point for each swollen and red toe, midfoot, digit, or knuckle and 5 points for each swollen ankle or wrist for a maximum of 60 per rat. Randomization of rats to treatment groups was completed in which every other rat that developed signs of disease on a given day was placed in the same treatment group (PEG-HCC or vehicle), thereby removing differences in basal disease severity on the day each rat developed signs of disease and accounting for differences in the time between pristane injection and when a rat developed signs of disease. PEG-HCC was given subcutaneously at 2 mg/kg every-other-day in the scruff of the neck and sterile saline was used as vehicle. This dose and route of administration of PEG-HCCs were selected from pharmacokinetic studies [16]. Age-matched healthy control animals were housed under the same conditions for the same duration. No animals were excluded during data analysis.

### 2.3. Cells

FLS cell lines from patients with RA or OA (Table 1), defined according to the criteria of the American College of Rheumatology [25] were developed from de-identified tissues obtained by the Feinstein Institute Tissue Donation program under and IRB-approved protocol [26,27]. PIA-FLS and healthy DA rat FLS were isolated from synovial tissues as described [28]. Human and rat FLS were cultured as described [26,27,28,29].

### 2.4. Internalization of PEG-HCCs by RA-FLS

To quantify cellular uptake of PEG-HCCs [16,17], FLS were incubated in ultralow adhesion 24-well plates with PEG-HCCs for different durations at 37 °C, 5% CO_2_. Cells were fixed with PBS + 1% paraformaldehyde. They were washed with flow cytometry buffer (PBS + 2% goat serum + 2% bovine serum albumin) and were either left intact or permeabilized with flow cytometry buffer + 0.5% saponin. Saponin was selected as the detergent as Tween-20 and Triton X-100 contain PEG and can produce false-positive signals. Cells were then stained for PEG-HCCs with anti-PEG antibodies (Table 2) and secondary goat anti-rabbit IgG antibodies before analysis by flow cytometry. Data were acquired on a FACSCanto II (Becton Dickinson, Franklin Lakes, NJ, USA) with the FACSDiva software and analyzed with the FlowJo software (FlowJo, LLC, Ashland, OR, USA).

### 2.5. Quantification of Intracellular Superoxide

RA-FLS or OA-FLS (10^4^ cells/200 µL/well) were allowed to adhere in a flat-bottom 96-well plate in medium + 10% FBS. Cells were loaded with 25 µg/mL DCFDA (Invitrogen, Carlsbad, CA, USA) for 45 min and washed before the addition of 1 µg/mL PEG-HCCs or vehicle. Fluorescence was detected 2 h later using a Tecan Infinite 200Pro plate reader with excitation at 490 nm and detection at 520 nm.

### 2.6. Cytotoxicity Assays

We assessed the effect of PEG-HCCs on inducing RA-FLS apoptosis using 7-AAD staining, as described [29,30]. RA- and OA-FLS were plated in 24-well plates and treated with 0.5 μM staurosporine (EMD Millipore, Burlington, MA, USA) or various concentrations of PEG-HCCs for 72 h. Cells were collected and stained with 7-AAD to determine the percentage of dead and dying cells, with detection by flow cytometry. Data were acquired on a FACSCanto II (Becton Dickinson, Franklin Lakes, NJ, USA) with the FACSDiva software and analyzed with the FlowJo software (FlowJo, LLC, Ashland, OR, USA).

### 2.7. Cell Proliferation Assays

We used incorporation of [^3^H] thymidine in the DNA of dividing cells to assess FLS proliferation, as described [23,29]. Briefly, FLS were plated into flat-bottom 96-well microplates (10^4^ cells/well) and incubated with vehicle (saline) or PEG-HCCs for 72 h at 37 °C, 5% CO_2_. [^3^H] thymidine (1 μCi/well) was added during the last 16–18 h of culture before DNA harvesting onto glass fiber filters. The amount of [^3^H] thymidine incorporated into DNA was determined with a β scintillation counter. 

### 2.8. Invasion Assays

The invasive properties of FLS were determined using Matrigel-coated transwell systems (BD Biosciences, San Jose, CA, USA), as described [23,29,31]. In brief, FLS were seeded in serum-free medium in the upper compartment of the inserts. For in vitro treatment, vehicle (saline) or PEG-HCCs were added to the cells and the lower compartment was filled with media + 10% FBS. After a 24-h incubation at 37 °C, 5% CO_2_, the invasive cells on the underside of the inserts were stained with crystal violet and counted under an inverted microscope (Olympus IX71).

### 2.9. Gelatin Gel Zymographies

FLS (5 × 10^4^ cells/300 μL/well) were cultured for 24 h in 24-well plates in the presence of vehicle (saline) or PEG-HCCs. We used gelatin gel zymography (Invitrogen, Carlsbad, CA, USA) to measure the production of matrix metalloprotease-2 in the culture supernatants, as described [29,32].

### 2.10. Bead Array-Based Quantification of MMPs and Cytokines

The secretion of IL-6, IL-8, MCP1, MMP-1, MMP-2, MMP-3, MMP-9, and VEGF by RA-FLS were determined using the RayPlex Custom Human Multiplex Bead Array Kit (Ray Biotech, Norcross, GA, USA), according to the manufacturer’s instructions. RA-FLS (5 × 10^4^ cells/400 μL medium/well) were treated with 1 µg/mL PEG-HCCs for 16–18 h. Culture medium was then collected and 25 μL of it was used for the detection of each analyte. Data were acquired on a FACSCanto II (Becton Dickinson, Franklin Lakes, NJ, USA) with the FACSDiva software and analyzed with the FlowJo software (FlowJo, LLC, Ashland, OR, USA).

### 2.11. Detection of PEG-HCCs by Immunohistochemistry

Rats received a single subcutaneous injection of 2 mg/kg PEG-HCCs or vehicle in the scruff of the neck 10 days after onset of clinical signs of PIA. Age-matched healthy rats were used as controls. They were euthanized 24 h later for collection of the hind limbs, fixation in 10% buffered formalin, paraffin embedding, and sectioning. Sections were stained for the detection of PEG-HCCs and podoplanin as described [16,33]. Briefly, sections were dewaxed in xylenes, rehydrated through an ethanol gradient, and non-specific binding sites were blocked overnight with PBS + 5% goat serum + 5% bovine serum albumin. BloxALL was used to block endogenous peroxidase and alkaline phosphatase. Slides were then incubated with anti-PEG antibodies (Table 2) for 2 h. After washes, single-stained slides were incubated with goat anti-rabbit IgG conjugated to horseradish peroxidase before detection with the Vector SG horseradish peroxidase substrate. For double-staining, sections were then incubated with anti-podoplanin or anti-CD3 antibodies (Table 2), followed by horse anti-mouse IgG conjugated to alkaline phosphatase before detection with the Vector red alkaline phosphatase substrate. 

### 2.12. Quantification of Circulating Rheumatoid Factor

Rat blood was collected by cardiac puncture [34] immediately prior to cardiac perfusion and euthanasia. Circulating levels of rheumatoid factor were measured by ELISA (Antibodies-online, Atlanta, GA, USA) in serum, following manufacturer’s instructions. 

### 2.13. X-rays, Histology, and Immunohistochemistry on Hind Limbs of Rats with PIA

At the end of the PIA trials, rats were deeply anesthetized with inhaled isoflurane for cardiac perfusion with saline [35], followed by decapitation to ensure death. Paws were immediately collected and either used for radiographic imaging (Bruker In-Vivo Xtreme) or fixed, decalcified, paraffin-embedded, and sectioned. Sections were stained with hematoxylin and eosin or with Safranin O-Fast Green [22,23]. Scoring of disease parameters, including synovial hyperplasia cartilage erosions, pannus extensions, and immune infiltrates were determined from the hematoxylin and eosin and Safranin O-Fast Green-stained sections by an investigator blinded to treatment groups, as previously described [36].

### 2.14. Phenotyping of T Lymphocyte Populations

To quantify T lymphocytes, inguinal and popliteal lymph nodes were collected from PIA rats after cardiac perfusion and euthanasia and single cell suspensions were prepared using cell strainers (70 µm). Cells were stained with a panel of antibodies as described [37], in which the cells were washed with flow cytometry buffer before staining with allophycocyanin-conjugated anti-rat CD3 antibodies (Becton Dickinson, 557030), phycoerythrin-cyanine-7-conjugated anti-rat CD4 antibodies (Biolegend, 201516), phycoerythrin-conjugated anti-rat CD8 antibodies (Becton Dickinson, 554857), fluorescein isothiocyanate-conjugated anti-rat CD25 antibodies (Becton Dickinson, 554865), and biotin-conjugated anti-rat CD45RC antibodies (Invitrogen, MA5-17458), followed by Alexa Fluor 405-conjugated streptavidin (Invitrogen, S32351). Cell fluorescence was measured by flow cytometry, as described above.

### 2.15. Statistics

All statistical analyzes were performed with the GraphPad software (San Diego, CA, USA). Two-way ANOVA with Bonferroni’s post-test was used to compare differences in clinical scores of PIA. One-way ANOVA with Dunnett’s test was used for FLS uptake of PEG-HCCs. All other assays were analyzed by One-way ANOVA with Tukey’s test. *p* values less than 0.05 were considered to be significant and data are presented as mean ± SEM.

## 3. Results

### 3.1. PEG-HCCs Are Internalized by RA-FLS and Reduce the Cells’ Intracellular O_2_^•−^ Levels

**Figure 1:**
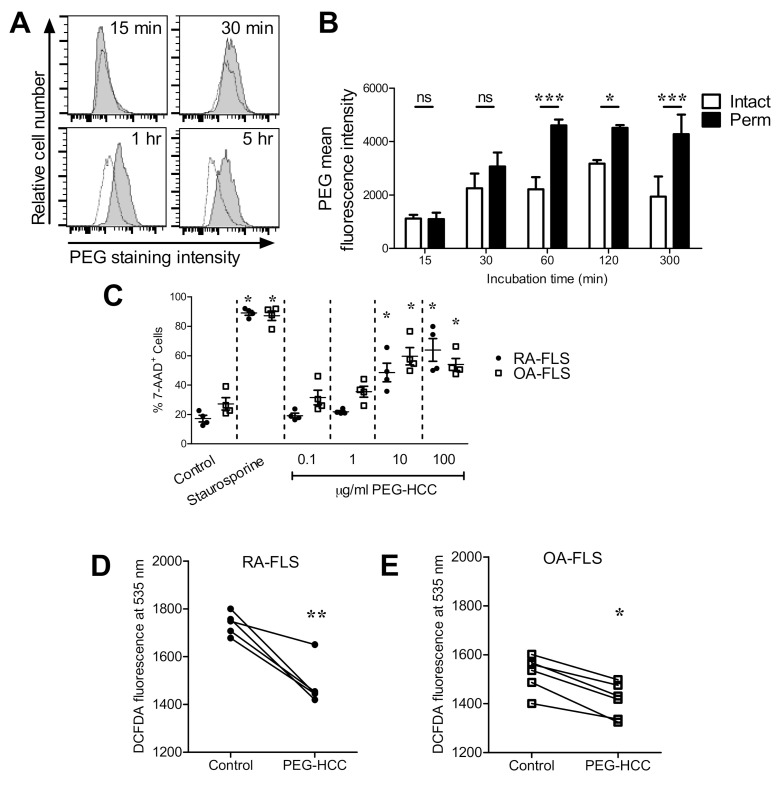
Poly(ethylene glycol)-functionalized hydrophilic carbon clusters (PEG-HCCs) are internalized by RA-FLS and reduce intracellular superoxide. (**A**) Example flow cytometric histograms of human RA-FLS treated with PEG-HCCs for various times, permeabilized, and stained against PEG (shaded) or unstained (white). (**B**) Quantification of PEG-HCCs in RA-FLS treated for various times with PEG-HCCs, left either intact or permeabilized, and stained for PEG expression (*n* = 3). (**C**) Measure of cytotoxicity through 7-AAD staining of RA-FLS (●, *n* = 4) and OA-FLS (□, *n* = 4) treated with staurosporine or PEG-HCCs for 72 h. Representative flow cytometry histograms are shown in Supplemental Figure S1. (**D**,**E**) DCFDA fluorescence of RA-FLS and OA-FLS treated with or without 1 μg/mL PEG-HCCs for 2 h (*n* = 5 RA-FLS and 6 OA-FLS; each data point represents cells from a different donor). * *p* < 0.05, ** *p* < 0.01, *** *p* < 0.001. Data are presented as mean ± SEM.

We first examined if PEG-HCCs enter RA-FLS in vitro and found that PEG-HCCs enter RA-FLS within 60 min of incubation at 37 °C (Figure 1A,B). We next sought to determine the effects of PEG-HCCs on FLS survival and intracellular O_2_^•−^ levels. RA-FLS are highly metabolic and produce higher levels of intracellular O_2_^•−^ [18,38] and may therefore respond differently to an intracellular antioxidant than FLS from healthy individuals. However, healthy individuals do not undergo therapeutic joint surgery, it is therefore difficult to obtain healthy FLS. As control FLS, we and others [39,40,41] therefore use FLS from patients with osteoarthritis (OA) which are less aggressive than RA-FLS in terms of migration, invasiveness, and production of cytokine, chemokines and growth factors [42,43]. Concentrations of 10 μg/mL PEG-HCCs and higher induce the death of both RA-FLS and OA-FLS (Figure 1C). Treatment of RA-FLS and OA-FLS with 1 μg/mL PEG-HCCs reduces the amount of intracellular O_2_^•−^ (Figure 1D,E). 

### 3.2. PEG-HCCs Alter FLS Phenotypes In Vitro

**Figure 2:**
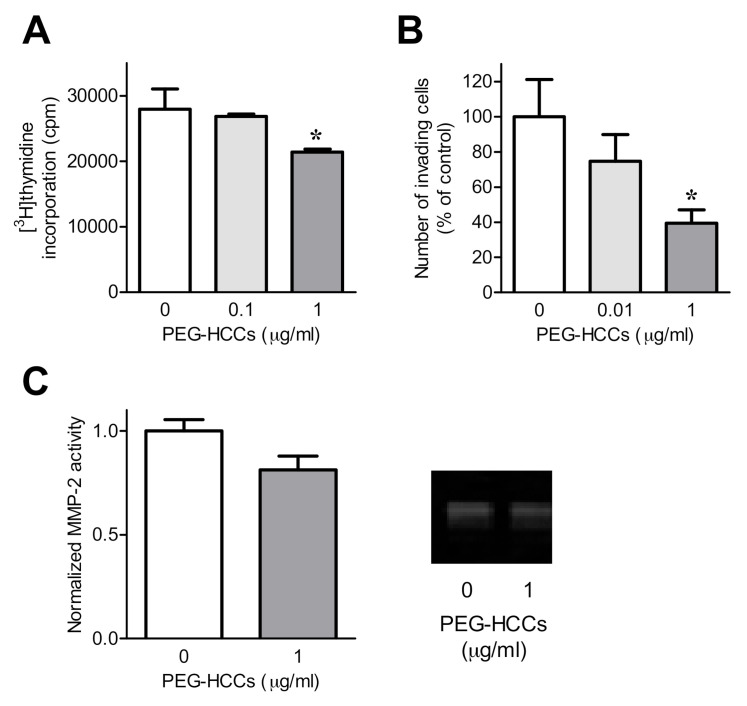
PEG-HCCs reduce RA-FLS invasion and proliferation. (**A**) Proliferation of RA-FLS treated with 0, 0.1, or 1 μg/mL PEG-HCCs for 72 h (*n* = 3). (**B**) Invasion through Matrigel-coated transwell inserts of RA-FLS in the presence of 0, 0.01, or 1 μg/mL PEG-HCCs (*n* = 3). Representative images are shown in Supplemental Figure S2. (**C**) Left, matrix metalloproteinase-2 (MMP-2) secretion of RA-FLS treated with or without 1 μg/mL PEG-HCCs for 24 h (*n* = 7). Right, example zymography gel of supernatants of RA-FLS treated with or without 1 μg/mL PEG-HCCs for 24 h. * *p* < 0.05. Data are presented as mean ± SEM.

Given that PEG-HCCs can enter FLS and reduce intracellular O_2_^•−^ levels without affecting their survival at low doses, we next determined the effects of PEG-HCCs on FLS function. RA-FLS are more invasive and produce more pro-inflammatory cytokines and growth factors than do OA-FLS [42]. We therefore focused our analysis on the function of RA-FLS. Treatment of RA-FLS with 1 μg/mL PEG-HCCs reduces the amount of proliferation (Figure 2A) and invasion through Matrigel-coated transwell inserts (Figure 2B). However, PEG-HCCs did not affect RA-FLS secretion of matrix metalloprotease-2 by RA-FLS (Figure 2C, Supplemental Figure S3). Results were confirmed using a multiplex bead array, which showed no changes in RA-FLS secretion of IL-6, IL-8, MCP1, MMP-1, MMP-2, MMP-3, MMP-9, and VEGF (Supplemental Figure S4). 

### 3.3. PEG-HCCs Are Found in Synovial Cells during PIA

**Figure 3:**
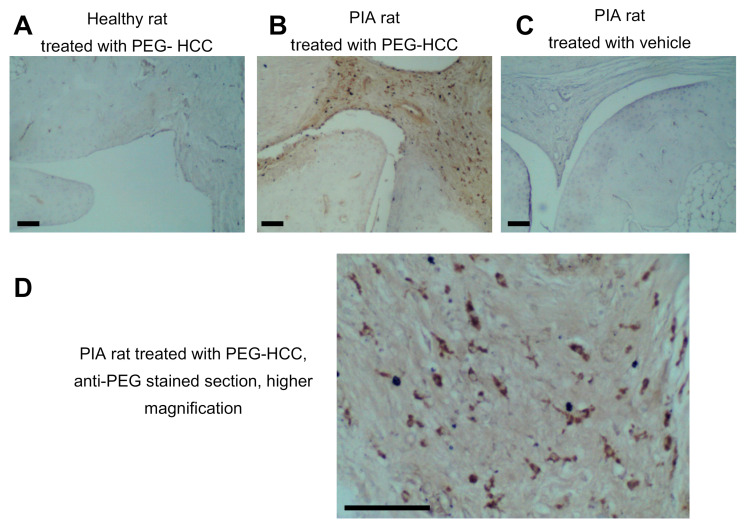
PEG-HCCs enter spindle-shaped cells in the synovium of rats with a model of RA. (**A**–**C**) Staining of PEG (brown) in the synovium of a healthy rat treated with PEG-HCCs (**A**), a PIA rat treated with PEG-HCCs (**B**), and a PIA rat treated with vehicle (**C**). (**D**) Higher magnification of a section from a PIA rat treated with PEG-HCCs and stained with anti-PEG antibodies. Scale bar = 100 μm.

Given our findings that PEG-HCCs can be internalized by primary human FLS and reduce their invasiveness, we sought to determine if PEG-HCCs reside within the synovial joints of either healthy rats or of rats with pristane-induced arthritis (PIA) following systemic administration of PEG-HCCs. Using an anti-PEG antibody that binds to conjugated but not to free PEG moieties [16], we detected PEG-HCCs within spindle-shaped cells residing in the synovium of hind paw joints of arthritic rats 24 h after PEG-HCC injection (Figure 3A–D). This staining was not detected in the synovium of healthy rats treated with PEG-HCCs or in the synovium of PIA rats treated with vehicle. 

### 3.4. PEG-HCCs Co-Localize with Podoplanin and CD3 in the Synovium of Rats with PIA

**Figure 4:**
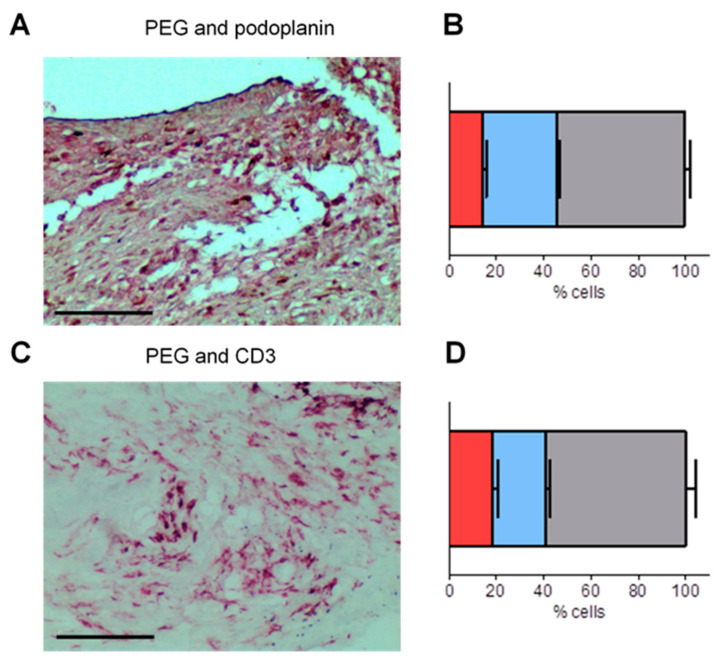
PEG-HCCs colocalize with podoplanin- and CD3-expressing cells. (**A**) Staining of PEG in blue/grey and of podoplanin in red in the synovium of a PIA rat treated with PEG-HCCs. Scale bar = 100 μm. (**B**) Quantification of cells expressing only podoplanin in red, of cells expressing only PEG in blue, and of cells expressing both markers in grey. (**C**) Staining of PEG in blue/grey and of CD3 in red in the synovium of a PIA rat treated with PEG-HCCs. Scale bar = 100 μm. (**D**) Quantification of cells expressing only CD3 in red, of cells expressing only PEG in blue, and of cells expressing both markers in grey. *n* = 6.

To further identify the cells that internalized the nanoparticles in the synovium, we double-stained tissue sections for detection of PEG-HCC and of either podoplanin, a marker of FLS during RA and its animal models [40,44,45] or CD3, a marker for T lymphocytes. We selected these two cell types because prior work showed that T lymphocytes endocytose PEG-HCCs [16] and we have shown above that human FLS can internalize PEG-HCCs in culture. The majority of podoplanin^+^ cells were also PEG^+^ (~78%, Figure 4A,B), as were the majority of CD3+ cells (~76%, Figure 4C,D), suggesting that both FLS and T lymphocytes internalize PEG-HCC in the synovium of rats with PIA. 

### 3.5. PEG-HCCs Reduce Disease Severity in PIA

**Figure 5:**
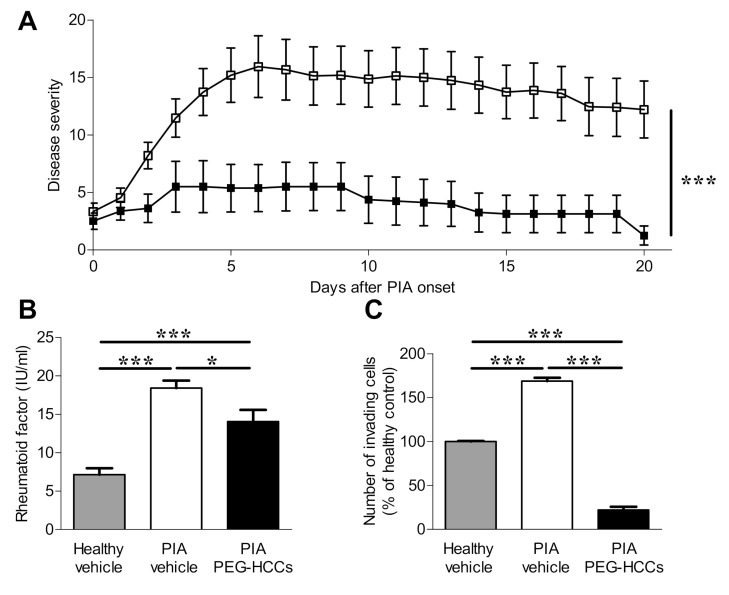
PEG-HCCs reduce disease severity in a rat model of RA. (**A**) Clinical scores of paw inflammation of rats with the PIA model of RA that were treated with vehicle (*n* = 15) or PEG-HCCs (*n* = 8) every-other-day for 21 days. (**B**) Rheumatoid factor concentrations in the circulation of healthy rats (*n* = 3), rats with PIA treated with vehicle for twenty days (*n* = 6), and rats with PIA treated with PEG-HCCs for twenty days (*n* = 7). (**C**) In vitro invasion through Matrigel of FLS isolated from healthy rats (*n* = 3), vehicle-treated PIA rats (*n* = 3), and PEG-HCC-treated PIA rats (*n* = 9). * *p* < 0.05, *** *p* < 0.001. Data are presented as mean ± SEM.

FLS and T lymphocytes both play a role in the pathogenesis of RA. As both cell types internalize PEG-HCCs and their functions are affected by the antioxidant, we assessed the effects of PEG-HCCs in the PIA rat model of RA. Rats with PIA were treated with either vehicle or PEG-HCCs every-other-day for 20 days starting on the day of disease onset. Vehicle-treated rats developed inflammation in multiple paw joints with maximum clinical scores of 15.2 ± 9.1 at peak of disease, 5 days after disease onset, whereas PEG-HCC-treated rats had scores of 5.4 ± 5.8, or 65% lower. At the end of the trials, 20 days after onset of clinical signs, vehicle-treated animals had scores of 12.2 ± 9.6. In contrast, at that time PEG-HCC-treated rats had clinical scores of only 1.3 ± 2.3, a reduction of approximately 90% compared to vehicle-treated rats with PIA (Figure 5A). 

Twenty days after disease onset, we collected serum and found that PEG-HCC-treated rats with PIA had significantly less circulating rheumatoid factor compared to vehicle-treated PIA rats (Figure 5B). We also found that FLS isolated from the paws of PIA rats that had been treated with PEG-HCCs were significantly less invasive through Matrigel-coated transwells than those isolated from vehicle-treated PIA rats (Figure 5C). 

### 3.6. PEG-HCCs Reduce Bone and Joint Damage in PIA

**Figure 6:**
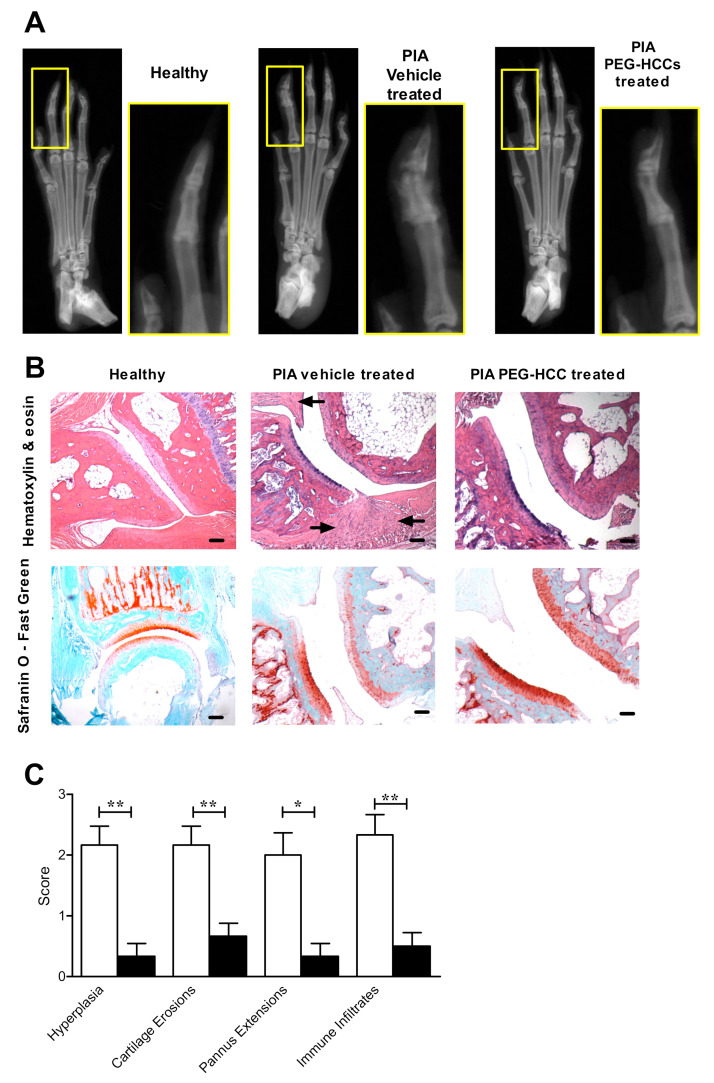
PEG-HCC-treated rats have reduced bone and synovial tissue destruction in PIA. (**A**) Example X-rays of the hind paws of a healthy rat, a vehicle-treated PIA rat, and a PEG-HCC-treated PIA rat. (**B**) Example images of hind paw joints of a healthy rat, a vehicle-treated PIA rat, and a PEG-HCC-treated PIA rat, stained with either hematoxylin & eosin (left) or safranin O-fast green (right). Arrows indicate areas of immune infiltrates in the synovium. Scale bar = 100 μm. (**C**) Quantification of pathologic hallmarks of disease of the joints of PIA rats treated with vehicle (white bars, *n* = 6) or PEG-HCCs (black bars, *n* = 6), as determined through analysis of hematoxylin & eosin and safranin O-fast green-stained joint sections. * *p* < 0.05, ** *p* < 0.001. Data are presented as mean ± SEM.

X-rays of the hind paws of rats with PIA from each treatment group twenty days after disease onset showed that PIA rats treated with PEG-HCCs had less bone destruction compared to vehicle-treated rats with PIA (Figure 6A). 

Histologic analyses of paw joints indicate that vehicle-treated rats with PIA had more synovial hyperplasia, cartilage destruction, pannus extensions, and immune infiltrates compared to the joints of healthy rats and of PIA rats that were treated with PEG-HCCs (Figure 6B,C). 

### 3.7. PEG-HCCs Do Not Induce Lymphopenia during PIA

**Figure 7:**
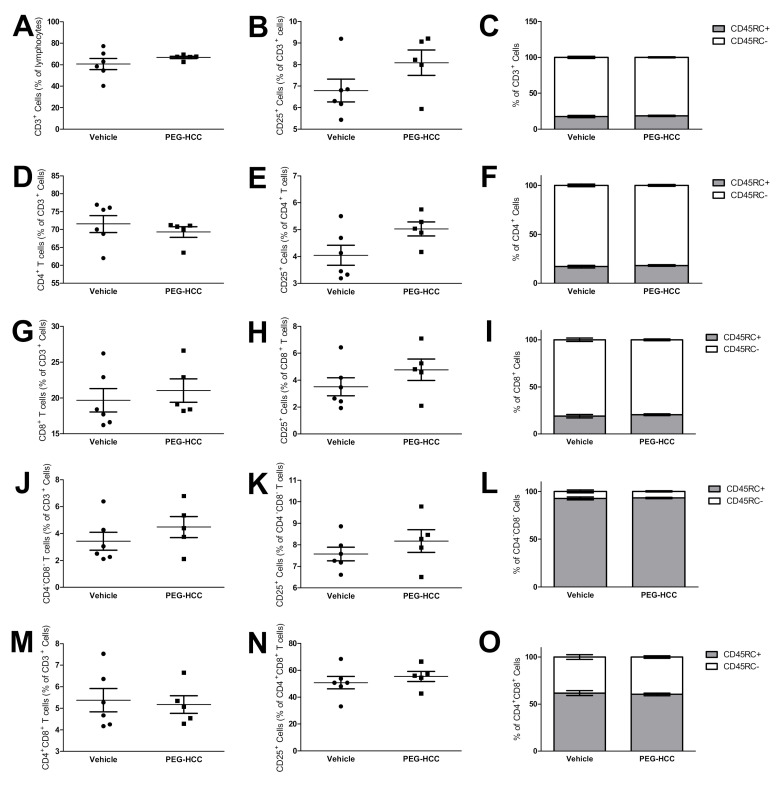
PEG-HCCs do not alter T cell populations in the lymph nodes during PIA. (**A**–**O**) Expression of CD3, CD4, CD8, CD25, and CD45RC of cells in the inguinal lymph nodes of rats with PIA treated with either vehicle (*n* = 6) or PEG-HCCs (*n* = 5) for twenty days after disease onset. Data are presented as mean ± SEM.

We have previously used flow cytometry and immunohistochemistry to show that PEG-HCCs are endocytosed by rat and human T lymphocytes but not by B or NK lymphocytes, dendritic cells, neutrophils, or macrophages [16]. As a result, PEG-HCCs do not affect phagocytosis, antigen processing and presentation, or fungistasis by macrophages [16]. Activators of antioxidant pathways, such as dimethyl fumarate, induce lymphopenia by killing activated T lymphocytes [46]. As T cells can endocytose PEG-HCCs [16,17], we collected the inguinal lymph nodes of vehicle-treated and PEG-HCC-treated PIA rats to determine if they too induce lymphopenia. We found no differences in the percentage of total T lymphocytes, identified by the expression of CD3 (Figure 7A), in the percentage of activated T cells (CD3^+^ CD25^+^) (Figure 7B), or in naïve (CD3^+^ CD45RC^+^) or memory (CD3^+^ CD45RC^−^) T lymphocytes (Figure 7C) [47]. Similar results were observed in CD4^+^ T cell populations (Figure 7C–F), CD8^+^ T cells (Figure 7G–I), CD4^-^CD8^-^ T cells (Figure 7J–L), and CD4^+^CD8^+^ T cells (Figure 7M–O). 

## 4. Discussion

In this study, we validated the use of PEG-HCCs as a potential novel therapeutic agent to reduce the pathogenic phenotype of FLS and the severity of an animal model of RA. In particular, we showed that PEG-HCCs reduce intracellular O_2_^•−^ levels, invasiveness, and proliferation of RA-FLS. We also provided evidence that the systemic administration of PEG-HCCs reduces disease severity in the PIA rat model of RA.

Intracellular ROS levels must be tightly controlled as either insufficient ROS from excessive amounts of PEG-HCC or excessive ROS from loss of O_2_^•−^ dismutase expression, induce FLS death [48]. Whereas in previous studies 100 µg/mL PEG-HCCs induced no toxicity in T lymphocytes [16], the nanoparticles induced the death of RA-FLS only at 10 µg/mL and higher concentrations. All subsequent in vitro assays were therefore done with doses of 1 µg/mL or lower. Pharmacokinetic studies of PEG-HCCs in rats showed that 2 mg/kg of PEG-HCCs given subcutaneously induces peak plasma concentrations of 0.5–0.7 µg/mL [16], well below the concentration that affects FLS viability. 

We have shown that PEG-HCCs enter RA-FLS, but the mechanisms for this entry remains to be determined. Nanoparticles have been shown to enter cells via active mechanisms, including endocytosis and phagocytosis [16]. After their endocytosis by T lymphocytes, PEH-HCCs localize inside the mitochondria [16]. Their intracellular localization inside FLS will also have to be examined. 

Whereas PEG-HCCs reduced the proliferation and invasiveness of RA-FLS, they did not affect the ability of these cells to secrete matrix metalloproteases. These findings suggest that the nanoparticles inhibit only some signaling pathways in the FLS. Furthermore, given that cellular invasion involves a complex interplay between cell migration and protease-mediated extracellular matrix degradation [49], our results indicate that the PEG-HCC-mediated reduction in RA-FLS invasion is likely due to PEG-HCCs altering components of cellular migration mechanisms, as opposed to protease production. The specific mechanisms by which PEG-HCCs alter RA-FLS migration, such as mediating adhesion molecule activation and localization and cytoskeleton arrangements, is yet to be determined. The mechanisms by which PEG-HCCs reduce RA-FLS proliferation will also need to be studied in more detail. 

FLS are not a homogeneous population in the synovium and can be separated in at least three subsets based on the expression of markers, their localization within the synovium, and their pathogenic roles during RA [40,45]. Furthermore, FLS vary from one joint to another [50]. A detailed analysis of the subsets of FLS able to internalize PEG-HCCs and affected by the nanoparticle will be needed. 

We found that PEG-HCCs were detectable in the synovial tissues of rats with PIA, but not in healthy rat synovium when given systemically. This was an intriguing finding and suggests that PIA-FLS have differential expression of proteins or membrane permeability that facilitate PEG-HCC uptake or that their increased metabolic activity during inflammation favors endocytosis, as was shown with some macromolecules [51]. In addition, the permeability of the blood-joint barrier is increased in inflamed joints [52], possibly allowing the entry of the nanoparticles into these joints. However, the mechanism by which PEG-HCCs selectively enter certain tissues and cells remains to be explored. Interestingly, treatment of rats with PIA with PEG-HCCs significantly reduced serum rheumatoid factor concentrations. We previously found that PEG-HCCs do not enter B lymphocytes [16], which produce rheumatoid factor, and it is therefore unlikely that PEG-HCCs directly affect rheumatoid factor production by B cells. Other immune cells, such as monocyte/macrophages, T lymphocytes, and dendritic cells can activate B cells. We have shown that PEG-HCCs are not internalized by monocyte or dendritic cells and do not affect antigen processing and presentation by macrophages, or their ability or phagocytose or kill fungi [16]. PEG-HCCs are endocytosed by T lymphocytes and inhibit their activation [16] and we show here that FLS, known to support B cell migration and survival [53], are affected by PEG-HCCs. It is possible that PEG-HCCs indirectly reduce the ability of B cells to produce rheumatoid factor by affecting T cells and/or FLS. 

We did not find differences in the relative composition of T cell populations in the lymph nodes of rats with PIA treated with either vehicle or PEG-HCCs. This result was unexpected as PEG-HCCs can inhibit the activation of T lymphocytes [16]. Though, while the percentage of T cell populations remained unchanged by PEG-HCCs, it is still possible that their activation was reduced and that PEG-HCC treatment reduced PIA disease severity at least in part through inhibiting T cells in addition to FLS. 

## 5. Conclusions

Overall, our studies suggest PEG-HCCs as a novel therapeutic option for the treatment of RA by scavenging O_2_^•−^ and inhibiting RA-FLS proliferation and invasiveness. 

## Figures and Tables

**Table 1:** Characteristics of rheumatoid arthritis–fibroblast-like synoviocytes (RA-FLS) and osteoarthritis–FLS (OA-FLS) donors.

Donor	Diagnosis	Sex	Ethnicity	Age	Disease Duration (Years)	RF	Medication(s)	Origin
OA-1	OA	Female	Caucasian	UN	UN	N/A	None	PSG
OA-2	OA	Female	African American	56	2	N/A	None	PSG
OA-3	OA	Female	Caucasian	56	5	N/A	None	PSG
OA-4	OA	Male	Caucasian	56	25	N/A	None	PSG
RA-1	RA	Female	Hispanic	64	>10	+	Prednisone, Methotrexate	PSG
RA-2	RA	Female	Caucasian	66	20	+	Prednisone, Etanercept	PSG
RA-3	RA	Male	Hispanic	48	<1	+	DMARD, Prednisone	Asterand
RA-4	RA	Female	African American	49	11	+	Prednisone, Plaquenil	PSG
RA-5	RA	Female	Caucasian	71	3	+	Methotrexate, Prednisone	PSG
RA-6	RA	Male	Caucasian	54	>10	+	Etanercept	PSG
RA-7	RA	Female	Caucasian	39	3	+	NSAID	Asterand

N/A: not applicable; UN: unknown; RF: rheumatoid factor; PSG: Dr. P.S. Gulko; DMARD: disease-modifying anti-rheumatic drug; NSAID: non-steroid anti-inflammatory drug.

**Table 2:** Antibodies Used.

Target	Host	Vendor (Location)	Catalog Number	Conjugation
**Primary Antibodies**				
PEG	Rabbit	Abcam	Ab51257	N/A
Rat CD3	Mouse	Becton Dickinson	557030	Allophycocyanin
Rat CD3	Mouse	Becton Dickinson	550295	N/A
Rat CD4	Mouse	Biolegend	201516	Phycoerythrin-cyanine-7
Rat CD8	Mouse	Becton Dickinson	554857	Phycoerythrin
Rat CD25	Mouse	Becton Dickinson	554865	Fluorescein isothyocyanate
Rat CD45RC	Mouse	Invitrogen	MA5-17458	Biotin
Rat/human podoplanin	Mouse	Abcam	Ab10288	N/A
**Secondary Antibodies**				
Rabbit IgG	Goat	Life Technologies	P-10994	Pacific Blue
Rabbit IgG	Horse	Vector Labs	MP-7401-50	Horseradish peroxidase
Mouse IgG	Horse	Vector Labs	AP-2000-1	Alkaline phosphatase
Streptavidin	N/A	Invitrogen	S32351	Alexa Fluor 405

## Supplementary Materials

The following are available online at https://www.mdpi.com/2076-3921/9/10/1005/s1, Figure S1. Representative flow cytometry histograms of 7-AAD staining shown in Figure 1C, Figure S2. Example of highly invasive RA-FLS pre and post invasion-suppressing treatments. Figure S3. Detection of MMP-2 by gelatin gel zymography. Figure S4. Detection of secreted proteins by RA-FLS following PEG-HCC treatment.
