# Supplementary material for: Antioxidant Carbon Nanoparticles Inhibit Fibroblast-Like Synoviocyte Invasiveness and Reduce Disease Severity in a Rat Model of Rheumatoid Arthritis
Source: Antioxidants (Basel). 2020 Oct 16;9(10):1005. doi: 10.3390/antiox9101005 (PMC7602875; doi:10.3390/antiox9101005)
Supplement: Supplementary file 1 [file antioxidants-09-01005-s001.pdf]

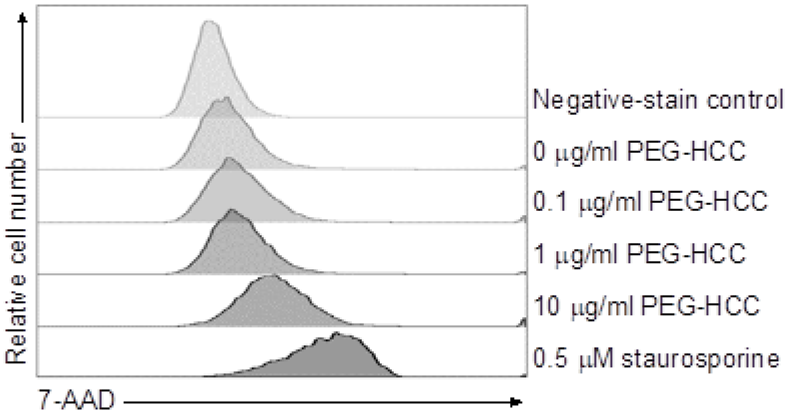

Figure 1. Representative flow cytometry histograms of 7-AAD staining shown in figure 1C. RA-FLS were treated for 72 hours with various concentrations of PEG-HCCs or 0.5 μM staurosporine prior to staining with 7-AAD and analysis by flow cytometry.

Highly-invasive RA-FLS RA-FLS with reduced invasiveness

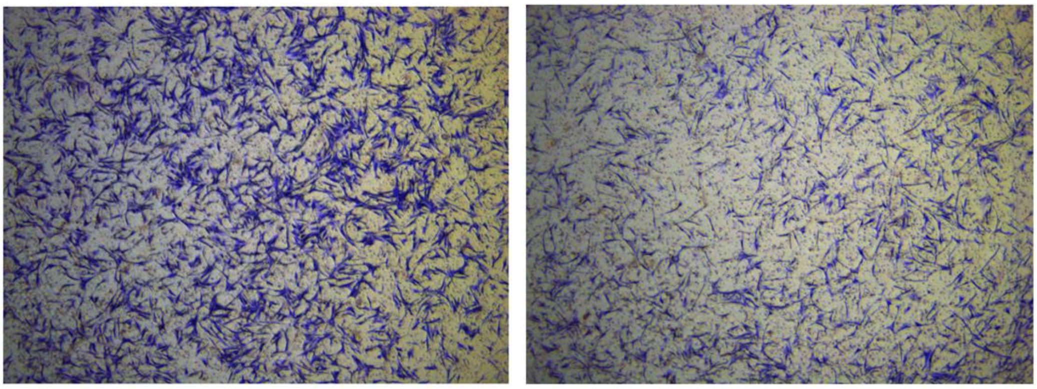

Figure 2. Example of highly invasive RA-FLS pre and post invasion-suppressing treatments. After a 24 hr invasion assay, cells were stained with crystal violet for counting.

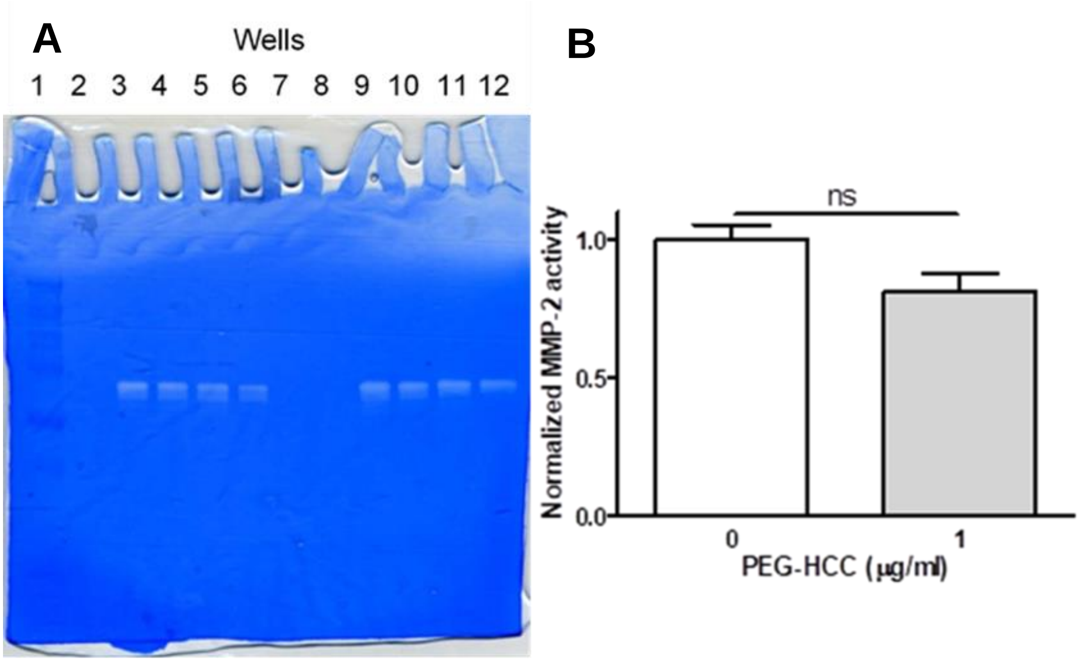

Figure 3. Detection of MMP-2 by gelatin gel zymography. A, representative gel showing the detection of MMP-2 in the supernatants of RA-FLS from two different donors. Wells and content: 1, molecular weight ladder; 2, empty, 3-6 RA-FLS donor 1; 7 and 8, empty; 9-12 RA-FLS donor 2. Supernatants in wells 3 and 9 contained no PEG-HCC. Supernatants from wells 4 and 10 contained 1 μg/ml PEG-HCC. Supernatants from wells 5 and 11 contained 10 μg/ml PEG-HCC. Supernatants from wells 6 and 12 contained 100 μg/ml PEG-HCC. **B,** MMP-2 secretion by RA-FLS treated with or without 0.1 or 1 μg/ml PEG-HCCs for 24 hours (n=7). Data with 10 μg/ml and 100 μg/ml PEG-HCCs were not plotted as the nanoparticles are toxic to RA-FLS at these doses. ns = not significant.

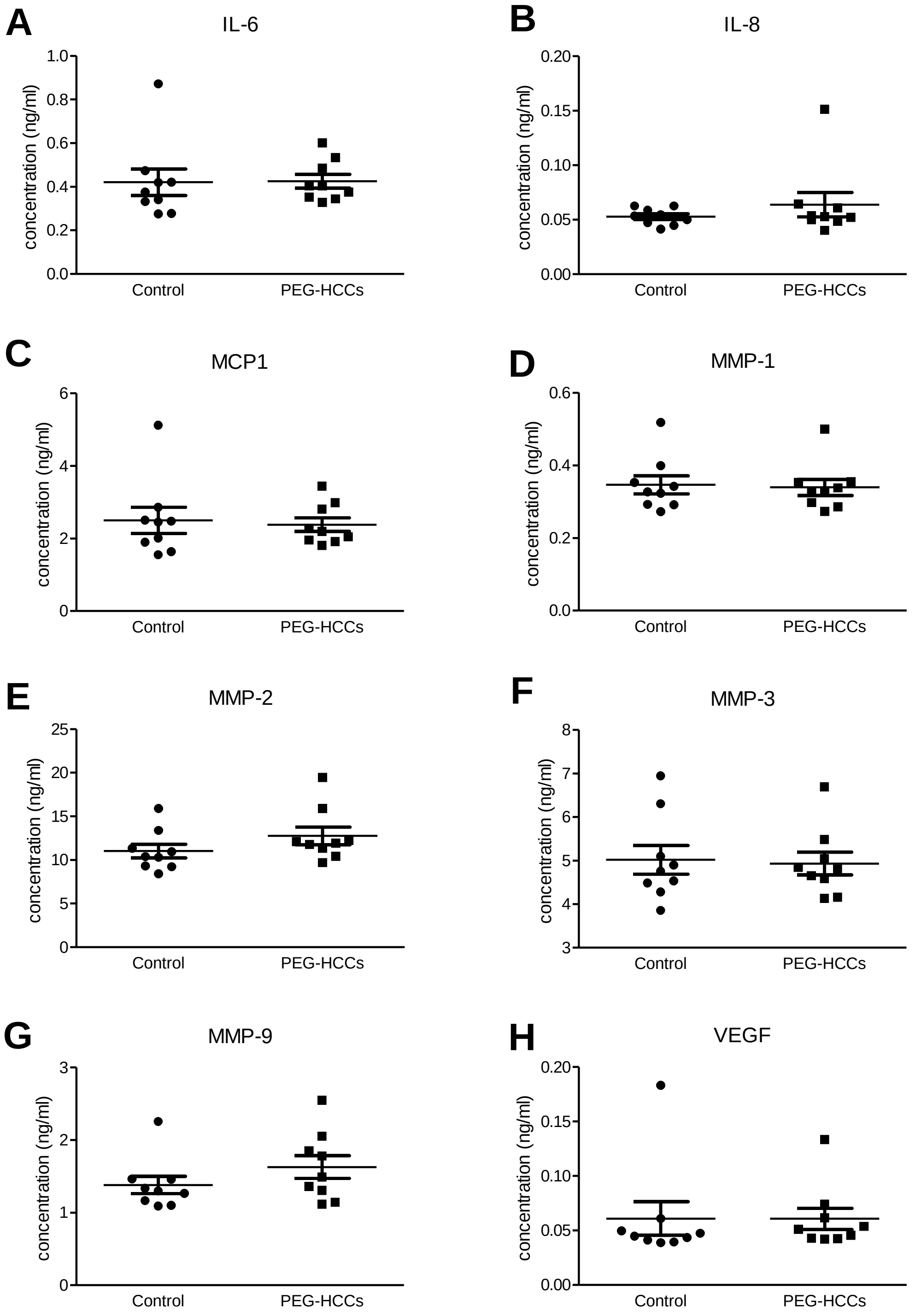

Figure 4. Detection of secreted proteins by RA-FLS following PEG-HCC treatment. A-H, Quantification of cytokines and proteases secreted by RA-FLS treated for 16-18 hours with 1 µg/ml PEG-HCCs, as measured by a multiplex bead array kit and detection by flow cytometry. n=9 RA-FLS donors.
